# Cognitive Performance on the Montreal Cognitive Assessment Test and Retinal Structural and Functional Measures in Glaucoma

**DOI:** 10.3390/jcm11175097

**Published:** 2022-08-30

**Authors:** Solmaz Bastani Viarsagh, Min Er Zhang, Sahar Shariflou, Ashish Agar, S. Mojtaba Golzan

**Affiliations:** 1 Vision Science Group, Graduate School of Health, University of Technology Sydney, Sydney 2007, Australia; 2 Department of Ophthalmology, Prince of Wales Hospital, University of New South Wales, Sydney 2031, Australia; 3 Marsden Eye Specialists, Sydney 2150, Australia

**Keywords:** cognitive decline, glaucoma, visual field reliability, retinal structure and function, retinal ganglion cell count estimates

## Abstract

**Background:** Glaucoma, the leading cause of irreversible blindness, is classified as a neurodegenerative disease, and its incidence increases with age. Pathophysiological changes, such as the deposition of amyloid-beta plaques in the retinal ganglion cell layer, as well as neuropsychological changes, including cognitive decline, have been reported in glaucoma. However, the association between cognitive ability and retinal functional and structural measures in glaucoma, particularly glaucoma subtypes, has not been studied. We studied the association between cognitive ability and the visual field reliability indices as well as the retinal ganglion cell (RGC) count estimates in a cohort of glaucoma patients. **Methods:** A total of 95 eyes from 61 glaucoma patients were included. From these, 20 were normal-tension glaucoma (NTG), 25 were primary open-angle glaucoma (POAG), and 16 were glaucoma suspects. All the participants had a computerised Humphrey visual field (HVF) assessment and optical coherence tomography (OCT) scan and were administered the written Montreal Cognitive Assessment (MoCA) test. RGC count estimates were derived based on established formulas using the HVF and OCT results. A MoCA cut-off score of 25 and less was designated as cognitive impairment. Student’s *t*-test was used to assess differences between the groups. The Pearson correlation coefficient was used to assess the association between MoCA scores and retinal structural and functional measures. **Results:** Significant associations were found between MoCA scores and the false-negative and pattern standard deviation indices recorded on the HVF (r = −0.19, r = −0.22, *p* < 0.05). The mean IOP was significantly lower in the cognitively impaired group (i.e., MOCA ≤ 25) (13.7 ± 3.6 vs. 15.7 ± 4.5, *p* < 0.05). No significant association was found between RGC count estimates and MoCA scores. Analysis of these parameters in individual glaucoma subtypes did not reveal any group-specific significant associations either.

## 1. Introduction

Glaucoma is the leading cause of irreversible blindness worldwide, characterised by peripheral vision loss accompanied by optic neuropathy with retinal ganglion cell (RGC) degeneration, retinal nerve fibre layer (RNFL) thinning, and optic disc cupping [1,2,3,4,5]. Attributable to cerebral cortex damage, glaucomatous optic neuropathy (GON) has also been observed in early cognitive decline [6]. While previous studies suggest that visual complications precede cognitive decline, the underlying mechanisms that link glaucoma and neurodegenerative diseases remain unclear [4,7]. To date, no study has explored the association between cognitive ability (quantified on a cognitive assessment scale) and retinal structural and functional measures in glaucoma patients. Cognitive decline may impact patients’ performance on the structural or functional measures of glaucoma. It may also be directly linked to pathophysiological changes associated with the disease. In either case, cognitive dysfunction may not only affect an individual’s quality of life but, more importantly, may adversely impact their clinical management.

Differences in the measurement scales and algorithms of retinal structural and functional changes in glaucoma have led to contradictory reports of such changes in glaucoma progression [1,3,8]. Nevertheless, when both structural and functional parameters are combined to assess neuronal loss, RGC count estimates have been shown to identify progressive glaucomatous damage [1,3,5,8]. Medeiros et al., conducted the first observational cohort study which found improved accuracy and reliability of glaucoma progression estimates using RGC count compared with isolated measures [3]. Their findings were later confirmed by Hirooka et al., who emphasised the adoption of combined structural and functional assessments, with better glaucoma progression detection rates observed with RGC count compared with SAP and OCT alone [1]. However, the impending pathophysiological link between cognitive ability and GON highlights the need to study the potential impact of cognitive impairment on the reliability of visual field (VF) assessment and RGC count estimates in glaucoma.

There are contradictory findings in the literature on the correlation between cognitive decline and VF reliability criteria [9,10]. Honjo studied 94 eyes of 51 glaucoma patients aged over 75 and observed no correlation between the Mini-Mental State Examination (MMSE) scores and VF reliability [9]. In contrast, Raman demonstrated that, in 113 eyes of 60 glaucoma patients aged over 50, cognitively impaired glaucoma patients are 2.4 times more likely to yield unreliable VF [10]. This study reported that every one-point decline in the clock drawing test (CDT) equates to overestimating the mean deviation (MD) by 0.1 dB [10]. The use of different cognitive screening tools such as CDT or MMSE may explain the contradictory findings reported by these studies. In comparison to CDT and MMSE, the Montreal Cognitive Assessment (MoCA) provides a highly accurate and objective measure of cognitive decline, as it examines several aspects of cognitive ability [9,10,11,12,13]. In this study, we investigated the association between cognitive ability (assessed using the MoCA test) and VF reliability indices as well as RGC count estimates. The outcomes of this study will provide further evidence on the link between GON and cognitive function and the need to consider cognitive ability when evaluating retinal structural and functional measures in glaucoma.

## 2. Materials and Methods

### 2.1. Participants

This study included 95 eyes of 61 glaucoma patients that met the inclusion criteria (36 females with an average age of 74 ± 9 years and 25 males with an average age of 70 ± 17 years). Participants were recruited from two subspecialty private glaucoma ophthalmology clinics in Sydney, Australia, over a 12-month period. All participants answered a general questionnaire including questions on basic demographics as well as their relevant medical history. The MoCA test was then administered. MoCA is scored on a scale of 0–30 and evaluates cognitive measures to evaluate performance based on seven cognitive domains: language, abstraction, orientation, memory, attention, naming, and visuospatial function [14,15]. Following this, ocular-specific measures including IOP (Goldmann), visual field assessment (Humphrey Visual Field (HVF) using the 24-2 SITA standard protocol (Zeiss, Germany)), and the RNFL thickness measured by OCT (Heidelberg Spectralis, Heidelberg, Germany) were recorded. RGC count was estimated using the methodology proposed by Medeiros which is based on the results obtained from OCT and HVF. The accuracy of RGC count estimates relies on the OCT and HVF assessments performed concurrently. As only a subset of 37 participants had both an HVF and OCT test during their visit, RGC count estimates were only calculated for this cohort. To mitigate the impact of learning effects associated with visual field testing, only patients that had undergone at least three visual field tests prior to this study were included. The mean arterial pressure (MAP) and the mean ocular perfusion pressure (MOPP) were also calculated.

Ethical approval was obtained from the University of Technology Sydney’s Human Research Ethics Committee. Written consent was obtained from all participants prior to any data collection and all examination protocols adhered to the tenants of the Declaration of Helsinki.

### 2.2. Glaucoma Diagnosis

Clinical glaucoma was diagnosed by a glaucoma subspecialist (A.A.). Our cohort included 20 normal-tension glaucoma (NTG) patients, 25 primary open-angle glaucoma (POAG) patients, and 16 glaucoma suspect patients. Of these, 39 eyes were diagnosed as primary open-angle glaucoma (POAG), 36 eyes as normal-tension glaucoma (NTG), and 20 eyes were glaucoma suspects. POAG was defined by elevated IOP > 21 mmHg with open anterior chamber angles, characteristic glaucomatous structural abnormalities at the optic nerve head, positive RNFL irregularities corresponding to optic nerve head changes, and corresponding HVFA defects (a cluster of contiguous points of visual field deficit at the *p* < 0.05 level, HVF outside normal limits, and/or standard pattern deviation with *p* ≤ 5%). NTG was defined as having congruent optic nerve and visual field anomalies in the presence of normal IOP defined as 10–21 mmHg. Glaucoma suspects were described as having normal HVF results, with a suspicious appearance of the optic disc or RNFL changes suggestive of glaucoma, with or without a family history of glaucoma. An abnormal IOP was not a standalone inclusion criterion for glaucoma suspects. Individuals with secondary glaucoma or comorbidities that had a potential effect on the glaucoma classification or had established diagnoses of cognitive impairment (CI) were excluded from this study.

### 2.3. Data and Statistical Analysis

A commonly used MoCA cut-off score was used to define cognitive impairment (CI) [11,12]: participants with a MoCA score less than (or equal to) 25 (out of max 30) were classified as cognitively impaired.

The mean arterial pressure (MAP) and the mean ocular perfusion pressure (MOPP) were also calculated using the following equations:

MAP = Diastolic Pressure + 1/3(Systolic Pressure − Diastolic Pressure)

MOPP = 2/3(MAP − IOP)

Comparisons between two independent groups were assessed using Student’s *t*-test and the Mann–Whitney U test. The D’Agostino–Pearson test was used to evaluate normality. For comparing three independent groups, the Kruskal–Wallis test was used. The relationship between MoCA scores and the visual field reliability indices, as well as structure–function measures, were analysed using the Spearman or Pearson correlation coefficient. All statistical analyses were completed using the GraphPad Prism software (San Diego, CA, USA).

## 3. Results

The average age for all 61 participants was 72 ± 9 years. Of those, 29 (47.5%) were found to be cognitively impaired (CI), having scored 25 or less on the MoCA test. Comparing the ocular and systemic physiological measurements (i.e., IOP, systolic and diastolic blood pressure, MAP, and MOPP) between CI and non-CI participants showed that only IOP was significantly lower in the CI group (13.7 ± 3.6 vs. 15.7 ± 4.5 mmHg, *p* = 0.01). The age between CI and non-CI patients was not significantly different either. Similarly, comparisons of the measurements based on the glaucoma subtype showed that IOP was significantly different between the three groups (*p* < 0.001). No statistically significant difference was observed in the MoCA scores between these three groups (*p* = 0.5) (Table 1).

### 3.1. Visual Field Reliability and Structure–Function Measures

To assess if patients with CI perform poorer on the HVF, VF reliability indices including false-positive rates (FP), false-negative rates (FN), duration, fixation loss (FL), mean deviation (MD), and pattern standard deviation (PSD) were compared between the CI and non-CI group. Our results demonstrated that there were no significant differences in any of the indices measured between the two groups. Comparing these parameters based on glaucoma subtypes revealed an expected significant difference in pattern standard deviation amongst the three groups (*p* < 0.05). Furthermore, we observed no significant differences in the RNFL thickness and RGC count estimates between the groups (*p* > 0.05) (Table 2). 

### 3.2. MoCA Was Associated with Visual Field Reliability Indices

Finally, we investigated if MoCA scores are correlated with VF reliability indices or RGC count estimates. Across the study population, a negative correlation was found between MoCA scores and test duration, FP, FN, PSD, FL, and RNFL. However, this correlation was statistically significant only between MoCA and PSD (r = −0.22, *p* = 0.03). Additionally, an association was observed between MoCA scores and FN, with lower MOCA scores correlated with higher FN (r = −0.19, *p* < 0.05). We observed no statistically significant correlations between MoCA scores and RGC count estimates or the RNFL thickness (*p* = 0.2, *p* = 0.5, respectively) (Table 3). 

## 4. Discussion

In this study, we investigated the association between cognitive ability (quantified using the MoCA test score) and the visual field reliability indices as well as structural and functional measures in glaucoma. A significant correlation was found between MoCA scores, false-negative rates, and pattern standard deviation on the VF. However, using a MoCA cut-off score of 25 to establish CI [11,12], and to compare the various measures between CI and non-CI, we observed no statistically significant difference between the two groups. Considering the ocular physiological parameters between CI and non-CI, we found IOP to be significantly lower in the CI group. We also observed the highest IOP in glaucoma suspects.

Glaucoma progression is believed to follow a three-stage continuum [16], where RGC dysfunction leads to RGC death, followed by structural changes and functional loss. However, recent evidence suggests that this is not the course of the disease for all patients [17]. The accurate determination of structural or functional changes relies heavily on the accuracy of the criteria used to define such change [17]. Nevertheless, our cohort included mainly mild-to-moderate patients, and evidence suggests that the OCT and HVF assessments are reliable tools to evaluate disease severity in this stage [18].

The association between CI and VF reliability indices has been studied before [9,10,14,19,20]. In a study conducted by Ramadan et al., an increase in the production of unreliable VF by 2.4 times was observed in CI patients. This study demonstrated that CI patients are more likely to produce higher FN, leading to an overestimation of MD by 0.1 dB [10]. However, when we examined the correlation between the MoCA and visual field reliability indices across our study population, we only found a significant and negative correlation between MoCA scores and PSD and FN. These results do not clinically represent a reduction in VF reliability. These contradictory findings may be due to the different cognitive tests used to examine cognitive function. The association between CI and decreased VF reliability can be explained by an increase in VF variability over time. Diniz et al., reported an increase in VF variability in a 2.5-year follow-up study during which they were able to monitor the progression of cognitive impairment and its effect on VF indices [14]. Considering that glaucoma and CI are evolving conditions, our study was not longitudinal to capture the VF variability at various stages of the diseases, and we did not have information on the duration since diagnosis for our participants; therefore, we can only speculate that our participants were not diagnosed with severe cognitive impairment to a degree that it affects the variabilities in the visual field indices leading to unreliable VF. Further research is necessary to investigate the longitudinal changes in CI and VF reliability in glaucoma patients.

Investigating the association between MoCA scores and measures of retinal function and structure showed no association between MoCA scores and RGC count estimates or the RNFL thickness. These findings are consistent with the results reported by Oktem et al., who demonstrated no association between MoCA scores and the RNFL thickness [21]. However, it contradicts a study conducted by Kesler et al., that demonstrated that RNFL thinning is more prominent in cognitive impairment such as Alzheimer’s disease [22]. The non-significant findings of our study do not completely rule out the association between cognitive impairment and retinal functional and structural changes in glaucoma. Two main reasons can be assumed for this. First, our study relied on the MoCA test to evaluate cognitive function. While the test has been shown to perform superior to other existing methods [11,12,13,23], there is evidence that the MoCA cannot establish impairment in specific cognitive domains compared with the gold standard (i.e., neuropsychological testing) [23]. Second, our study cohort included patients at different stages of the disease spectrum. Previous studies have demonstrated a non-linear relationship between MD and RGC count estimates, whilst the RNFL thickness has a linear relationship with RGC count estimates [3,24]. This means that, for patients with early glaucomatous damage, an RGC count estimate corresponds to small changes in MD, but relatively larger changes in the RNFL thickness, whilst for patients at the advanced stages, the opposite is true (i.e., larger changes in MD and small changes in the RNFL thickness). Collectively, the association between CI and retinal functional and structural measures could be negatively implicated by the RGC count estimates relative to each individual patient’s disease stage. Further studies with a larger and homogenous patient cohort are required to further assess the relationship between cognitive impairment and retinal functional and structural measures.

We also investigated systemic (MAP) and ocular (IOP, MOPP) physiological parameters in our cohort. We found that the CI group had a significantly lower mean IOP compared with the non-CI group. Whilst this finding contradicts previous studies that demonstrate a significant association between increased IOP and accelerated neurodegenerative activity in RGCs, our findings are not completely surprising, mainly due to the fact that over 60% of the patients in the CI group were on IOP-lowering medications. Furthermore, we found that the mean MAP and MOPP in the CI group were non-significantly lower than those of the non-CI group. These findings are consistent with those reported in the current literature. Havenon et al., found that MAP is not significantly different in participants that have developed dementia, but rather it is the blood pressure variability that is correlated with cognitive impairment [15]. Similarly for MOPP, a study conducted by Szegedi et al., on 47 patients with mild cognitive impairment and 43 healthy controls [25] also showed a non-significant difference between the two groups (54.9 ± 7.4 mmHg vs. 53.3 ± 6.4, *p* = 0.31). The lower MAP and MOPP values in the CI group, once again, may be attributable to more than half the patients being on medication for hypertension and glaucoma.

## 5. Limitations

To the best of our knowledge, this is the first study to utilise the MoCA test to examine the association between cognitive decline and VF reliability as well as RGC count estimates in glaucoma patients. Nonetheless, our study has some limitations. First, we used MoCA scores solely to establish CI diagnosis, which might have affected our results because of its inability to establish impairment in specific cognitive domains. Additional examinations such as neuropsychological tests are necessary to confirm the diagnosis of CI. Second, the variations between follow-up appointment times, types of glaucoma eye drops, high blood pressure medications administered, and treatment compliance for each patient are potential confounders that were not controlled for. Finally, our sample size was small, and our study was not longitudinal. Furthermore, we were only able to measure RGC count estimates in a small subset of our cohort due to the requirement of concurrent OCT and HVF assessments. A larger and heterogeneous study population is required to further evaluate our findings for a longer period to capture the longitudinal changes in CI and VF in glaucoma patients since they are both evolving conditions.

## 6. Conclusions

In this study, we found no statistically significant association between cognitive ability (quantified using the MoCA test) and the visual field reliability indices or RGC count estimates (as a representative measure for combined retinal function and structure). Whilst a significant association between MoCA scores and false-negative and pattern standard deviation indices on the HVF was found, clinically, this does not establish an unreliable visual field result. Collectively, as our cohort included a diverse range of glaucoma patients at various stages of the disease, our findings do not completely rule out a potential link between cognitive ability and visual field reliability or retinal functional and structural measures. Rather, we propose that further studies are warranted, with larger cohorts and allowing comparison of patients with similar disease severity.

## Figures and Tables

**Table 1:** Participant demographics. Assessed based on (A) MoCA scores and (B) glaucoma subtypes.

	A			B			
	With CI MoCA ≤ 25	Without CI MoCA > 25	*p*-Value	Suspect	POAG	NTG	*p*-Value
n	29	32	--	16	25	20	--
Gender (F/M)	16/13	20/12	--	7/9	17/8	12/8	--
Age	75 ± 8	71 ± 10	0.09	69 ± 9	75 ± 10	72 ± 7	0.14
MoCA	22.4 ± 2.2	27.7 ± 1.5	<0.001	26 ± 4	24.7 ± 3.2	25.1 ± 2.7	0.5
IOP	13.7 ± 3.6	15.7 ± 4.5	0.01	16.5 ± 4.3	15 ± 3.4	13 ± 4.5	<0.001
Systolic	136 ± 17	145 ± 25	0.12	140 ± 24	142 ± 24	140 ± 18	0.9
Diastolic	84 ± 8	88 ± 12	0.19	87 ± 9	85 ± 10	86 ± 12	0.8
MAP	102 ± 9	107 ± 14	0.11	105 ± 13	104 ± 13	104 ± 12	0.9
MOPP	59 ± 6	61 ± 10	0.16	58 ± 9	59 ± 8	61 ± 8	0.4

MoCA—Montreal Cognitive Assessemnt. IOP—Intraocular Pressure. MAP—Mean Arterial Pressure. MOPP—Mean Ocular Perfusion Pressure.

**Table 2:** Distribution of visual field and structure–function characteristics. Assessed based on (A) MoCA scores and (B) glaucoma subtypes.

	A			B			
	MoCA ≤ 25	MoCA > 25	*p*-Value	Suspect	POAG	NTG	*p*-Value
False Positive (%)	2.8 ± 3.4	2.7 ± 2.7	0.9	2.8 ± 2.7	3 ± 3.7	2.4 ± 2.5	0.6
False Negative (%)	2.5 ± 3.8	2.5 ± 4.1	0.9	2.4 ± 4.3	2.2 ± 3.7	2.9 ± 4	0.7
MD (dB)	−4.4 ± 6.3	−4 ± 5.5	0.7	−1.4 ± 3	−4.9 ± 5.8	−5 ± 6	0.05
PSD (dB)	4.2 ± 3.7	4.3 ± 3.4	0.8	2.7 ± 2.2 *	4.1 ± 3.2	5.2 ± 4.1 *	0.04
Duration (sec)	345 ± 81	344 ± 71	0.9	330 ± 73	351 ± 81	345 ± 71	0.6
Fixation loss (%)	9.6 ± 10	7 ± 9	0.2	8.7 ± 11	8.9 ± 9.3	7.3 ± 9.2	0.7
RNFL (um)	80 ± 17	74 ± 12	0.1	82 ± 18	73 ± 13	79 ± 15	0.2
RGC count (×1000)	685 ± 203	687 ± 167	0.9	738 ± 195	635 ± 183	708 ± 177	0.1

POAG—Primary Open Angle Glaucoma. NTG—Normal Tension Glaucoma. MD—Mean Deviation. PSD—Pattern Standard Deviation. RNFL—Retinal Nerve Fibre Layer. RGC—Retinal Ganlion Cell. * Post-hoc analysis—significant difference was observed between the Suspect and NTG group.

**Table 3:** The association between MoCA Scores and visual field reliability and structure–function measures (all eyes included).

	Correlation Coefficient	*p*-Value
False positive (%)	0.04	0.6
False negative (%)	−0.19	0.04
MD (dB)	0.24	0.1
PSD (dB)	−0.22	0.03
Duration (sec)	−0.18	0.08
Fixation loss (%)	−0.18	0.07
RNFL (um)	−0.07	0.5
RGC (×1000)	0.13	0.2

## Data Availability

The datasets used and/or analysed during the current study are available from the corresponding author upon reasonable request.

## Abbreviations

MoCA	Montreal Cognitive Assessment
POAG	Primary open-angle glaucoma
NTG	Normal-tension glaucoma
RGC	Retinal ganglion cell
IOP	Intraocular pressure
HVF	Humphrey visual field
OCT	Optical coherence tomography
GON	Glaucomatous optic neuropathy
RNFL	Retinal nerve fibre layer
CI	Cognitive impairment
Aβ	Amyloid beta
MAP	Mean arterial pressure
MOPP	Mean ocular perfusion pressure
